# Multipaddled Anterolateral Thigh Chimeric Flap for Reconstruction of Complex Defects in Head and Neck

**DOI:** 10.1371/journal.pone.0106326

**Published:** 2014-09-02

**Authors:** Canhua Jiang, Feng Guo, Ning Li, Wen Liu, Tong Su, Xinqun Chen, Lian Zheng, Xinchun Jian

**Affiliations:** Department of Oral and Maxillofacial Surgery, Xiangya Hospital, Central South University, Changsha, Hunan, China; di Pompeo d'Illasi Sapienza, University of Rome, School of Medicine and Psychology, Italy

## Abstract

The anterolateral thigh flap has been the workhouse flap for coverage of soft-tissue defects in head and neck for decades. However, the reconstruction of multiple and complex soft-tissue defects in head and neck with multipaddled anterolateral thigh chimeric flaps is still a challenge for reconstructive surgeries. Here, a clinical series of 12 cases is reported in which multipaddled anterolateral thigh chimeric flaps were used for complex soft-tissue defects with several separately anatomic locations in head and neck. Of the 12 cases, 7 patients presented with trismus were diagnosed as advanced buccal cancer with oral submucous fibrosis, 2 tongue cancer cases were found accompanied with multiple oral mucosa lesions or buccal cancer, and 3 were hypopharyngeal cancer with anterior neck skin invaded. All soft-tissue defects were reconstructed by multipaddled anterolateral thigh chimeric flaps, including 9 tripaddled anterolateral thigh flaps and 3 bipaddled flaps. The mean length of skin paddle was 19.2 (range: 14–23) cm and the mean width was 4.9 (range: 2.5–7) cm. All flaps survived and all donor sites were closed primarily. After a mean follow-up time of 9.1 months, there were no problems with the donor or recipient sites. This study supports that the multipaddled anterolateral thigh chimeric flap is a reliable and good alternative for complex and multiple soft-tissue defects of the head and neck.

## Introduction

Though relatively uncommonly, complex soft-tissue defects of head and neck which involve multiple and nonadjacent anatomic sites can arise after radically surgery of advanced-staged malignancies or multiple lesions of the upper aerodigestive tract. Meanwhile, immediate flap reconstruction is the gold standard for surgical treatment for the defects after tumor ablation. However, functional and aesthetic reconstruction of complex and multiple defects in head and neck is still a major surgical challenge, because the reconstruction of multiple and nonadjacent defects demand a flexible and feasible flap with multiplanar configuration and multiple paddles [1]. There are several reconstructive choices open to the clinician, including local cutaneous flaps, pedicled fasciocutaneous flaps and microsurgical free flaps. It is usually not applicable for local and pedicled flaps to reconstruct such complex defects because of their limited soft-tissue amount and less versatile design, while using two or more individual flaps for multiple defects might be an opinion, but not the best. An ideal reconstructive procedure for multiple and complex three-dimensional defects should be performed in a single stage operation, which not only can reduce operative time but also prevent likelihood of postoperative complications.

For multiple and complex soft-tissue defects, reconstruction with three-dimensional microvascular flaps is often the preferred alternative. The free anterolateral thigh (ALT) flap, as first described by Song et al [2] in 1984, is a versatile and reliable reconstructive option. This flap has already been a workhorse flap for head and neck reconstruction in recent decades [3], [4]. The advantages of ALT flap include consistent and reliable anatomy, long vascular pedicle, the feasibility to create multiple skin paddles by recruiting additional perforators, the flexibility to reconstruct composite defects by recruiting different tissue types, and low donor site morbidity. Based on multiple cutaneous perforators originated from the descending branch of the lateral circumflex femoral artery (LCFA), free ALT flap can be harvested with multiple skin paddles for multiple and complicated soft-tissue defects, which can eliminate the need for two or more separately flaps and vascular anastomosis [5]–[7]. In this study, we present our experience with free multipaddled ALT chimeric flap for functional and aesthetical reconstruction of complex head and neck soft-tissue defects involving multiple nonadjacent anatomic sites in a single operation.

## Patients and Methods

Between May 2009 and August 2012, a total of 12 consecutive patients with head and neck cancer indicated for radical resection and complex soft-tissue reconstruction with multipaddled ALT chimeric flaps were enrolled. Of the 12 cases, 11 were men and only 1 was woman, with a mean age of 47.3 years (range, 33 to 62 years). None presented with distant metastases at the time of surgery. Tumor size was T4 in 4 patients (33.3%) and T3 in 8 cases (66.7%). The baseline data about 12 cases are shown in Table 1.

**Table 1:** Statistical description of case series.

No.	Age/Sex	Aetiology	TNM stage	Reconstructed area	Flap size (W × L cm)	Donor-site scar (W/L cm)	Follow-up (month)
1	46/M	Right BC with OSF	T3N1M0	Right through-and-through cheek	P: 4×6	0.7/25	8
					M: 6×9		
				Left buccal mucosa	D: 3×5		
2	34/M	Left BC with OSF	T4N2aM0	Left through-and-through cheek	P: 5×7	0.5/23	10
					M: 6×8		
				Right buccal mucosa	D: 2.5×4		
3	43/M	Left TC and BC with multiple leukoplakia	T3N0M0 (TC)	Right tongue mucosa	P: 3×5	1.0/26	12
			T2N0M0 (BC)	Left partial tongue	M: 5×7		
				Left buccal mucosa	D: 6×8		
4	56/M	Recurrent HC with neck skin invasion	T4N0M0	Pharyngoesophagus	P: 7×12	0.6/24	6
				Anterior neck skin	D: 7×8		
5	58/M	Recurrent Left BC with right buccal OSF scar	T3N0M0	Left through-and-through cheek	P: 7×8		
					M: 7×10		
				Right buccal mucosa	D: 3×5		
6	62/M	Left TC with OSF	T3N1M0	Left buccal mucosa	P: 3×6	0.8/22	18
				Left half tongue	M: 5×8		
				Right buccal mucosa	D: 3×5		
7	54/M	Right BC with OSF	T3N1M0	Right through-and-through cheek	P: 5×6	0.5/21	8
					M: 7×8		
				Left buccal mucosa	D: 4×4		
8	51/M	HC with neck skin invasion	T4N1M0	Pharyngoesophagus	P: 6×10	0.8/19	7
				Anterior neck skin	D: 5×7		
9	43/M	Left BC with OSF	T3N2aM0	Left through-and-through cheek	P: 4×7	1.2/22	10
					M: 6×8		
				Right buccal mucosa	D: 3×5		
10	54/M	HC with neck skin invasion	T4N1M0	Pharyngoesophagus	P: 6×9		
				Anterior Neck skin	D: 4×5		
11	33/F	Left BC with OSF	T3N1M0	Left through-and-through cheek	P: 5×7	0.9/20	14
					M: 6×9		
				Right buccal mucosa	D: 3×4		
12	34/M	Right BC with OSF	T3N0M0	Right through-and-through cheek	P: 3×6	0.6/22	8
					M: 7×9		
				Left buccal mucosa	D: 4×5		

Abbreviations: BC, buccal cancer; TC, tongue cancer; HC, hypopharyngeal cancer; OSF, oral submucous fibrosis; P, proximal paddle; M, middle paddle; D, distal paddle; W, width; L, length.

After radical ablation of cancer and other relative lesions, the resulting soft-tissue defects were classified into three types according to the locations of soft-tissue defects: type I, patient with advanced buccal mucosa cancer in one site and oral submucous fibrosis (OSF) of contralateral buccal mucosa (n = 7, Case 1, 2, 5, 7, 9, 12, 13); type II, patient with oral cancer and multiple oral mucosa lesions (leukoplakia or OSF) (n = 2, Case 3, 6); type III, patient with primary or recurrent hypopharyngeal cancer which has also invaded anterior neck skin (n = 3, Case 4, 8, 11).

The study followed the ethical guidelines of the Ministry of Health, China. Protocols applied in this study and the publish of patients' details have been approved by the Hospital Ethical Committee of the Xiangya Hospital. The individuals in this manuscript have given written informed consent to publish these case details.

### Reconstructive Procedures

All patients were examined preoperatively by using a portable Super Dopplex D900 non-directional hand-held Doppler probe (Huntleigh Diagnostics, Glamorgan, UK) to predict the vessel perforators originated from the branches of the lateral circumflex femoral artery on the left or right anterolateral thigh. Enough separately and suitable perforators should be detected on at least one thigh. Patients without enough perforators on both thighs were excluded. All defects were soft tissue. The relevant surgical technique has previously been described [8], [9]. Briefly, a line was drawn between the anterior–superior iliac spine and the midpoint of the lateral border of the patella on the donor thigh in supine position. The locations of main cutaneous perforators were detected using ultrasound Doppler preoperatively. Skin paddles were designed around the detected perforators. The pedicle of the multipaddled ALT flap was supplied by the descending branch or transverse branch of the lateral circumflex femoral vessels. A medial incision was made above the rectus femoris muscle and continued underneath the deep fascia of vastus lateralis to identify the located cutaneous perforators with intramuscular dissection. Perforators were then dissected in a retrograde fashion until arriving at the descending branch of the lateral circumflex femoral artery. For some cases, a small segment of vastus lateralis muscle around perforator was included as additional volume to augment the dead space and decrease tissue retraction after postoperative radiotherapy. And partial deep fascial cuff and soft-tissue around the perforator was left to prevent the vessel spasm and facilitate the fixation of perforator. The integrity of lateral cutaneous nerve was preserved carefully to decrease the risk of postoperative lateral thigh paresthesia. All skin paddles of multipaddled ALT flap was checked for viability and then transferred to the defect with vessel anastomosis. We usually select two recipient veins for anastomosis, which can secure the venous return of the flap. Hemostasis and drainage should maintained adequately to prevent hematoma formation and the flap should be monitored carefully by clinical examination for color and capillary refill during the early postoperative period. For excluded cases due to insufficient perforators, contralateral or bilateral ALT flaps could be supplementary to reconstruct all defects.

In our present study, for type I patients, after radical ablation, the bilateral complex defects involved full-thickness cheek on one side and contralateral buccal mucosa. Tripaddled ALT musculocutaneous flap was raised, and the proximal and middle paddles were chimeric and restored for the full-thickness cheek defects, while the distal paddle for the contralateral mucosa defect.

For type II patients, after surgical resection of tongue cancer and oral multiple lesions, resulting soft-tissue defects included unilateral or bilateral tongue and buccal mucosa. Tripaddled ALT musculocutaneous flap was used to reconstruction the 3-dimensional defects. The proximal and distal flaps were used to repair the bilateral mucosa defects respectively, and the middle one for tongue defect.

For type III patients, the invaded pharyngoesophagus and anterior neck skin were radical resected. Bipaddled ALT fasciocutaneous or musculocutaneous flap was raised. The larger skin flap was whole or near circumferential to reconstruct the pharyngoesophagus, while the remaining for the skin defect.

## Results

Initially, 13 cases were selected for this study. One was excluded due to no enough perforators intraoperatively, although Doppler showed enough perforator signals preoperatively in the operated thigh. For the remaining 12 cases, multipaddled ALT fasciocutaneous or musculocutaneous chimeric flaps were harvested to reconstruction complex 3-dimensional defects in head and neck. The mean length of skin paddle was 19.2 (range: 14–23) cm and the mean width was 4.9 (range: 2.5–7) cm. All flaps survived and all donor sites were closed primarily. After a mean follow-up time of 9.1 (5–18) months, there were no problems with the donor or recipient sites. All patients underwent postoperative radiotherapy.

### Case report

#### Case 1

**Figure 1:**
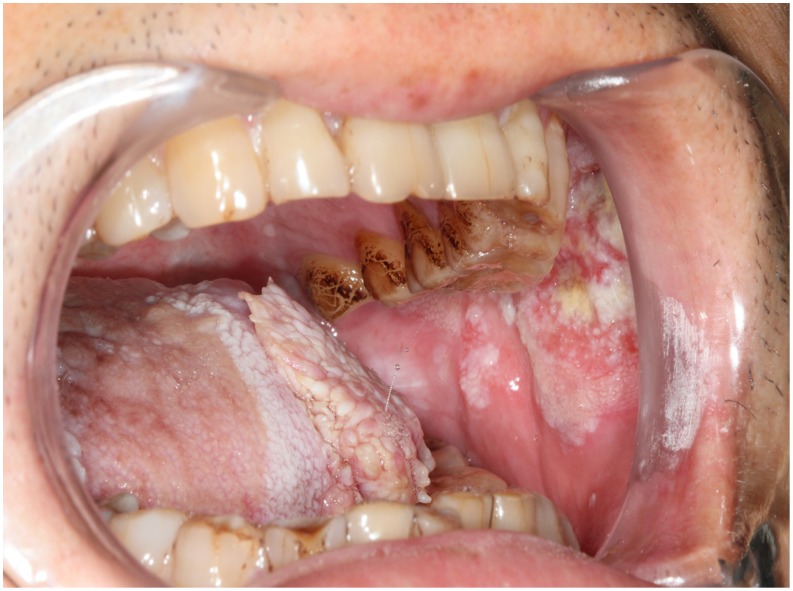
Preoperative view of two primary tumors. Note that the left tongue cancer (T3N0M0) and left buccal cancer (T2N0M0) were not directly adjacent.

**Figure 2:**
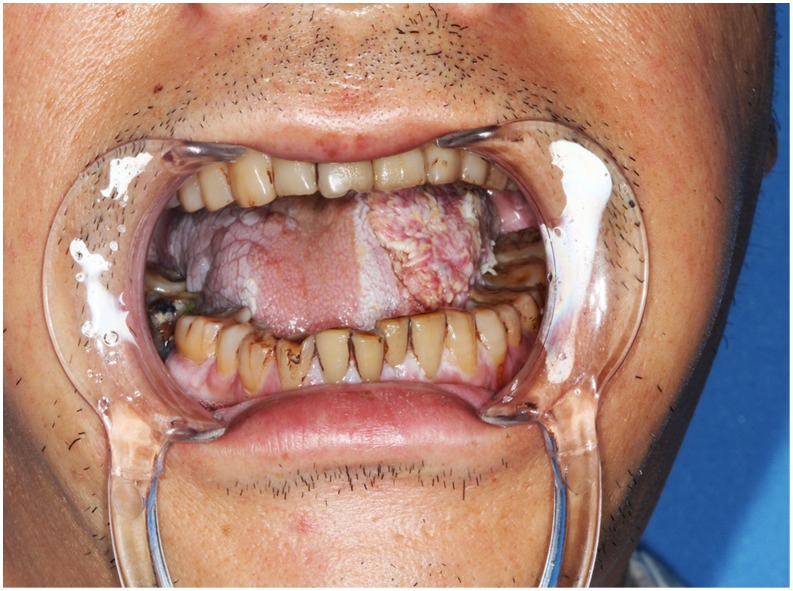
Preoperative view of left tongue cancer and right tongue mucosa leukoplakia. The extensive mucosa leukoplakia (3×4 cm^2^) of right tongue was indicated to be resected.

**Figure 3:**
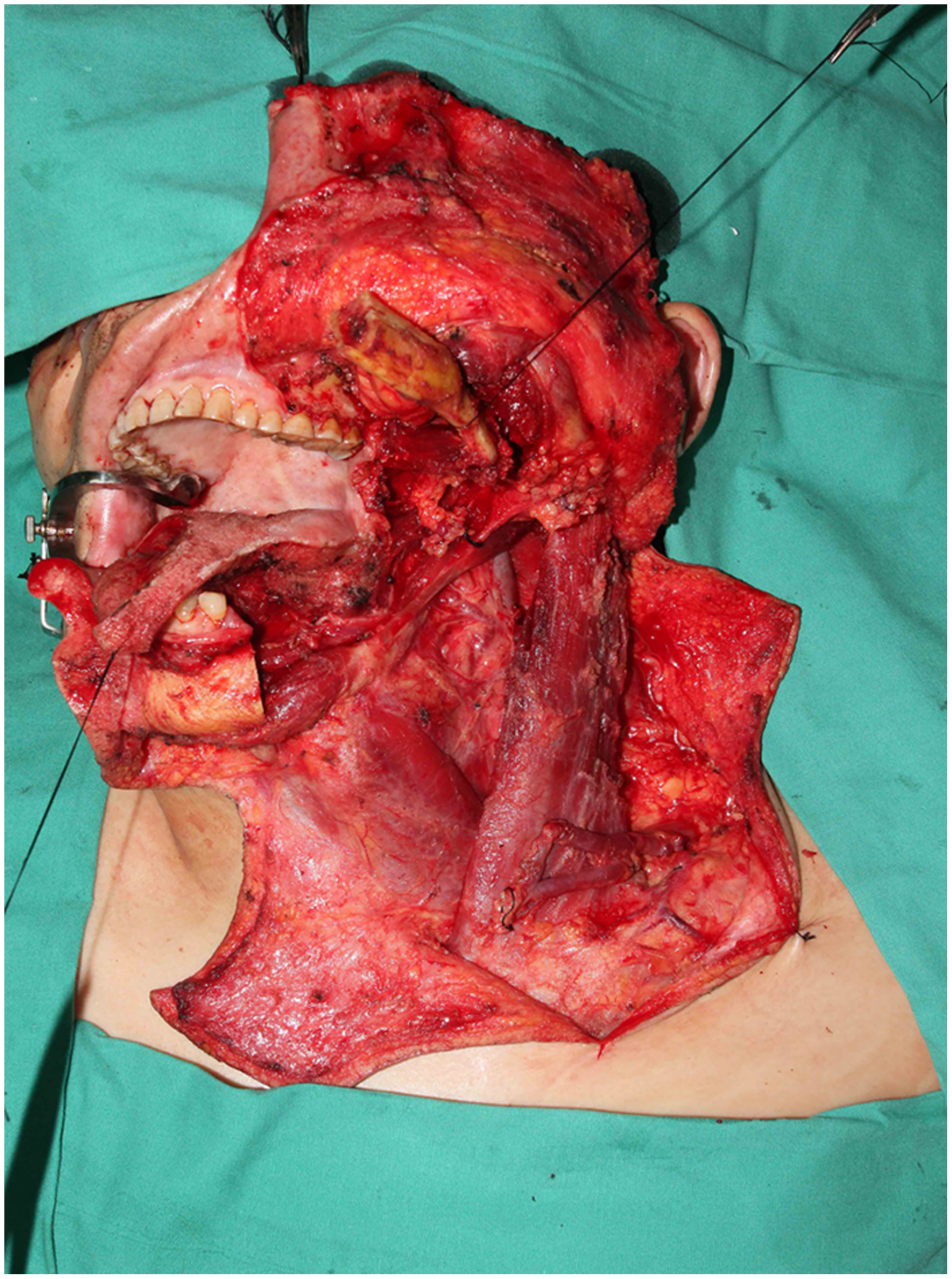
Intraoperative view of three individual defects involved left half tongue, left buccal mucosa, and right tongue mucosa after the radical cancer ablation.

**Figure 4:**
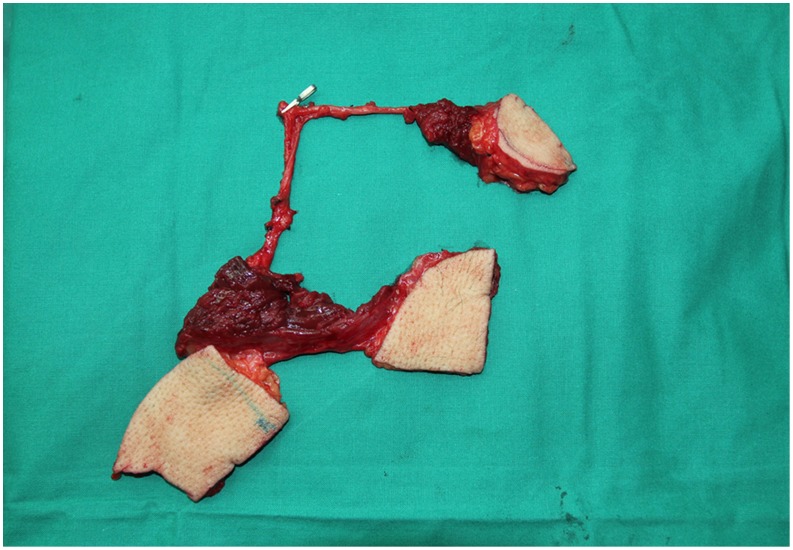
Intraoperative view after elevation of the tripaddled free ALT musculocutaneous flap.

**Figure 5:**
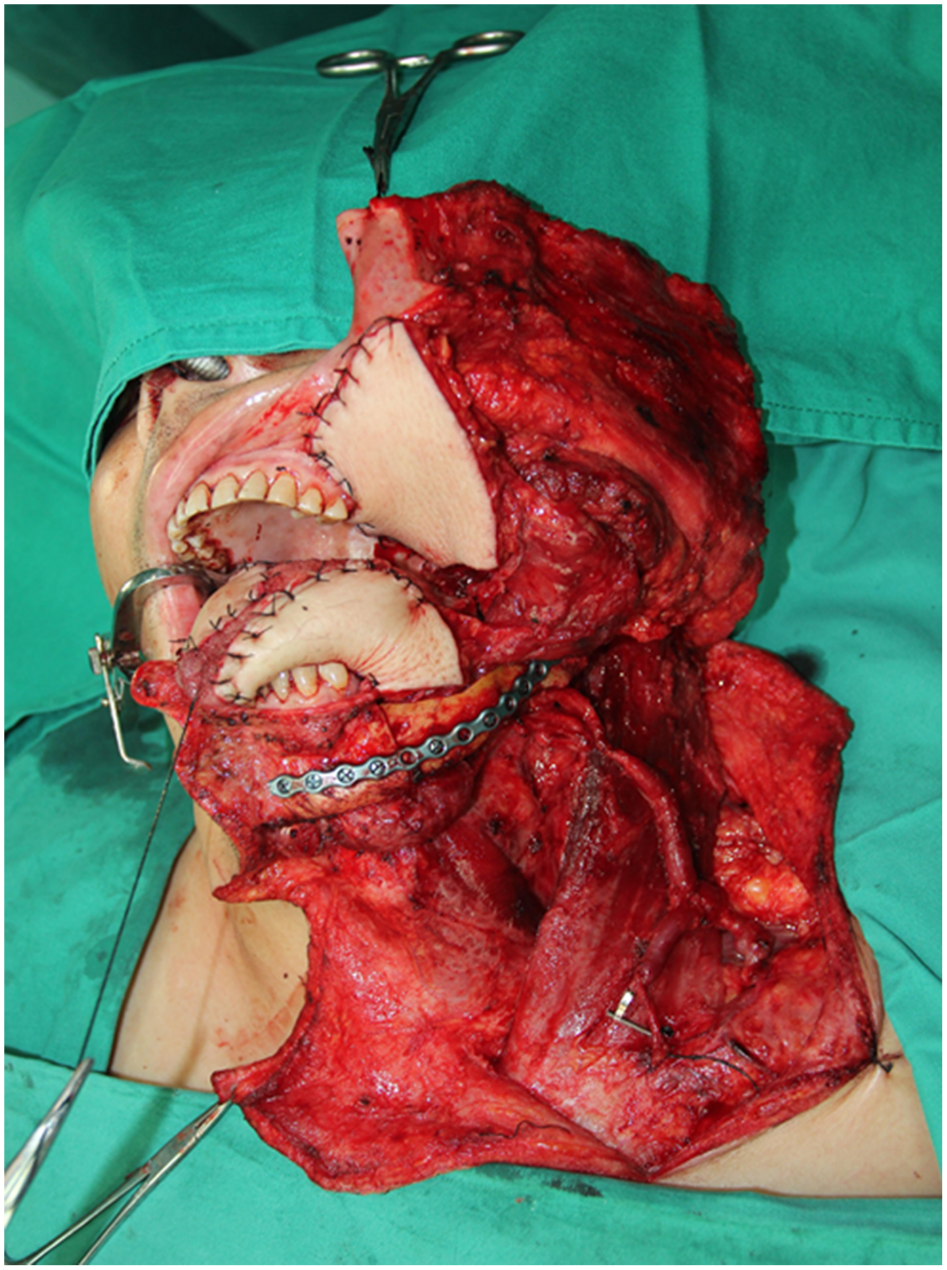
Intraoperative view of three separately skin paddles covering left buccal, left tongue, as well as right tongue mucosa defect respectively. Vascular anastomosis was made between the artery of the flap and the left superior thyroid artery and between the veins of the flap and the external jugular vein as well as a branch of internal jugular vein.

**Figure 6:**
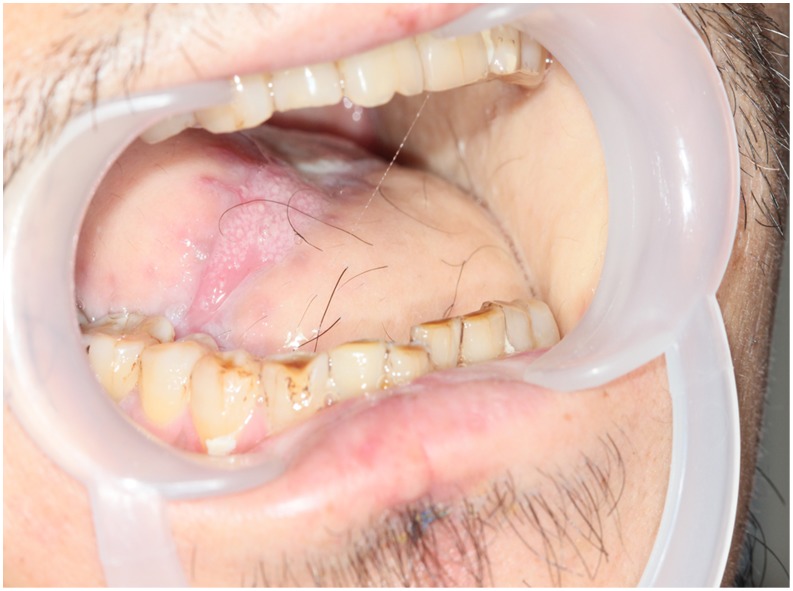
Appearance 2 months after operation with complete flap survival.

A 43-year-old man (case number 3 in Table 1) presented with left tongue cancer (T3N0M0), left buccal cancer (T2N0M0) and extensive mucosa leukoplakia on right tongue (Fig. 1 and 2) underwent a radical ablation of all oral lesions and left functional neck dissection. The resulting defects involved left half tongue, left buccal mucosa ranging from the gingival buccal sulcus of maxilla to left hyomandibular furrow of mandible, and right tongue mucosa (Fig. 3). One-staged reconstruction by tripaddled free ALT musculocutaneous flap was performed on the left thigh (Fig. 4). Partial vastus lateralis was included around the musculocutaneous perforator for augmentation of dead space. The distal skin paddle (6×8 cm^2^) was used to cover the left buccal defect, the middle one (5×7 cm^2^) for left half-tongue defect, and the proximal paddle (3×5 cm^2^) for right tongue defect (Fig. 5). The donor-site defect was closed primarily. Three skin paddles were all survival, and no recurrence occurred during 12-month follow-up (Fig. 6).

#### Case 2

**Figure 7:**
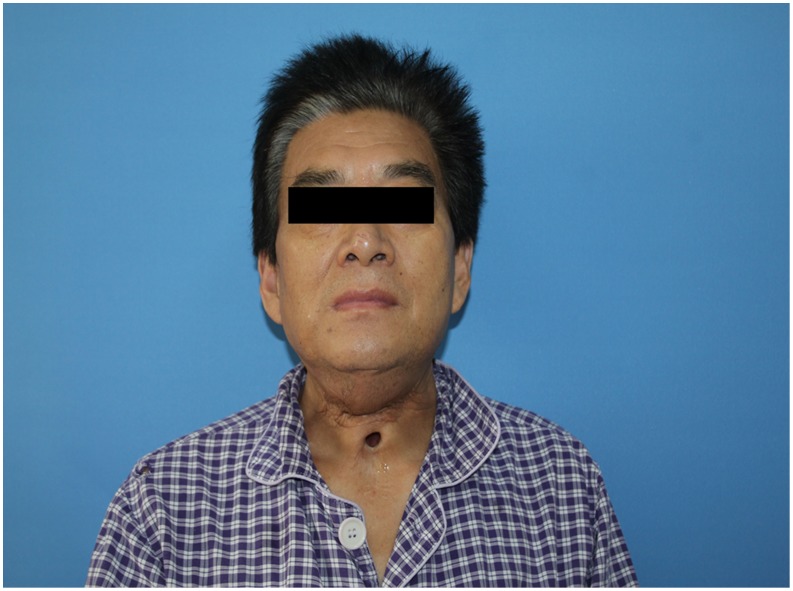
Preoperative view of a recurrent hypopharyngeal cancer invaded the anterior neck skin.

**Figure 8:**
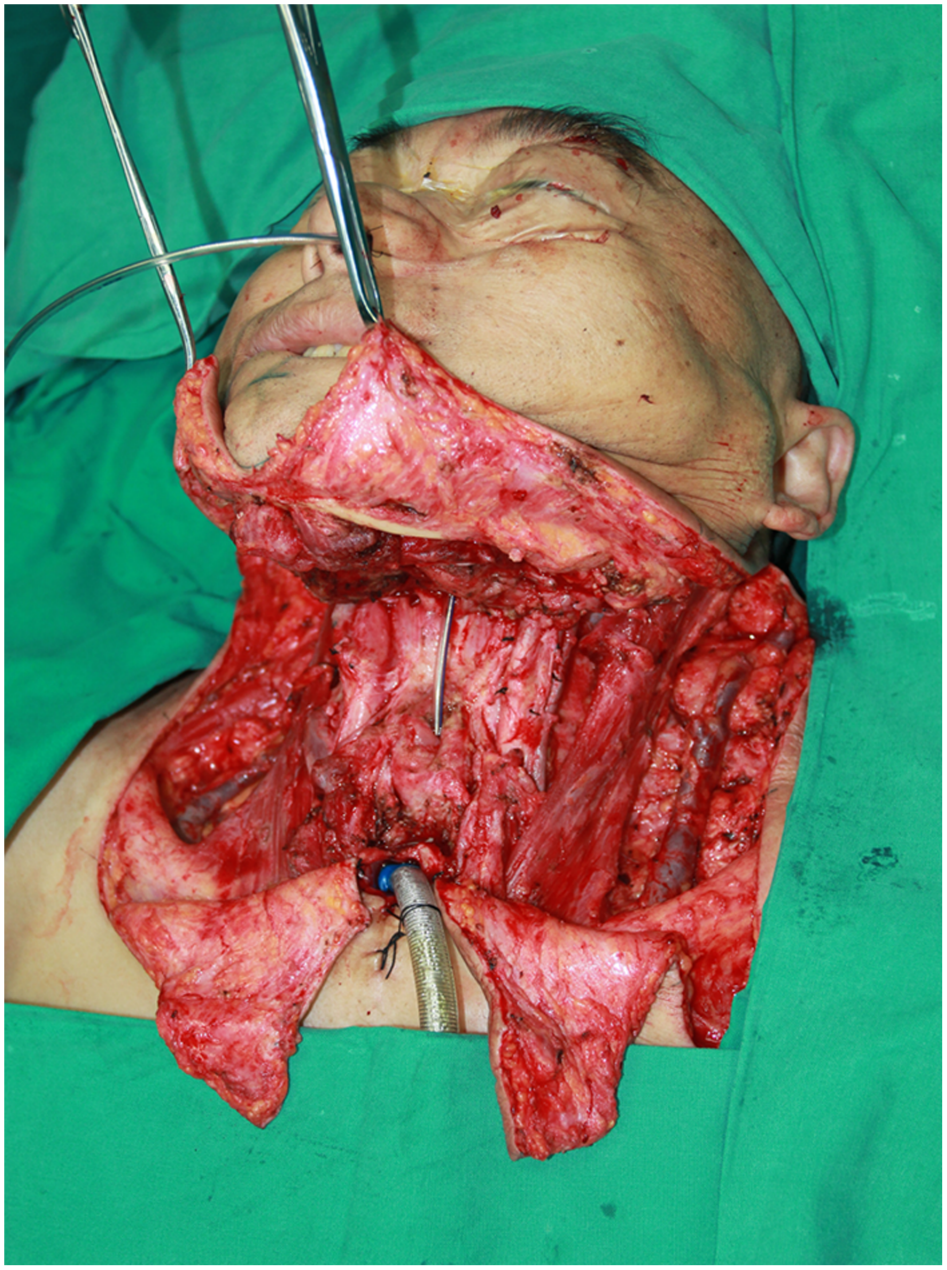
Intraoperative view of the resulting defects involved a part of esophagus and anterior neck skin after a radical salvage surgery including pharyngoesophagus, anterior neck skin and bilateral neck dissection.

**Figure 9:**
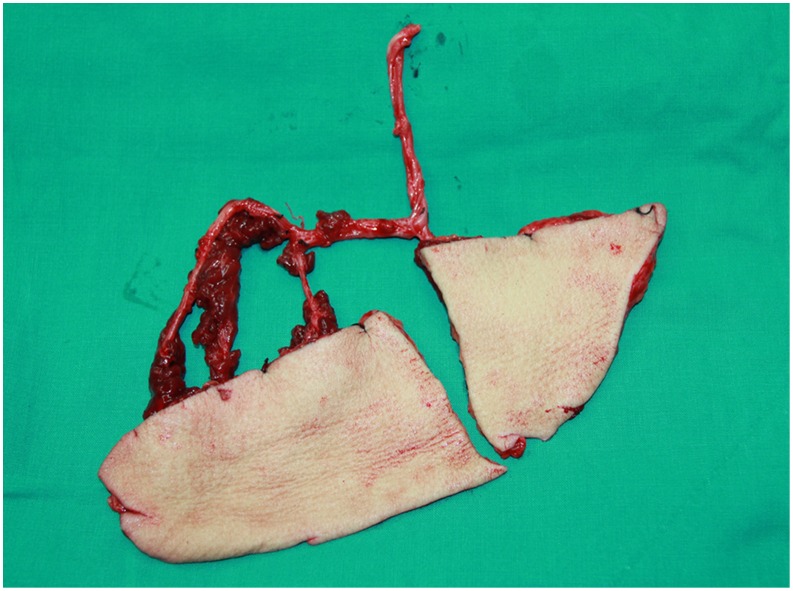
Intraoperative view after harvest of bipaddled free ALT fasciocutaneous flap.

**Figure 10:**
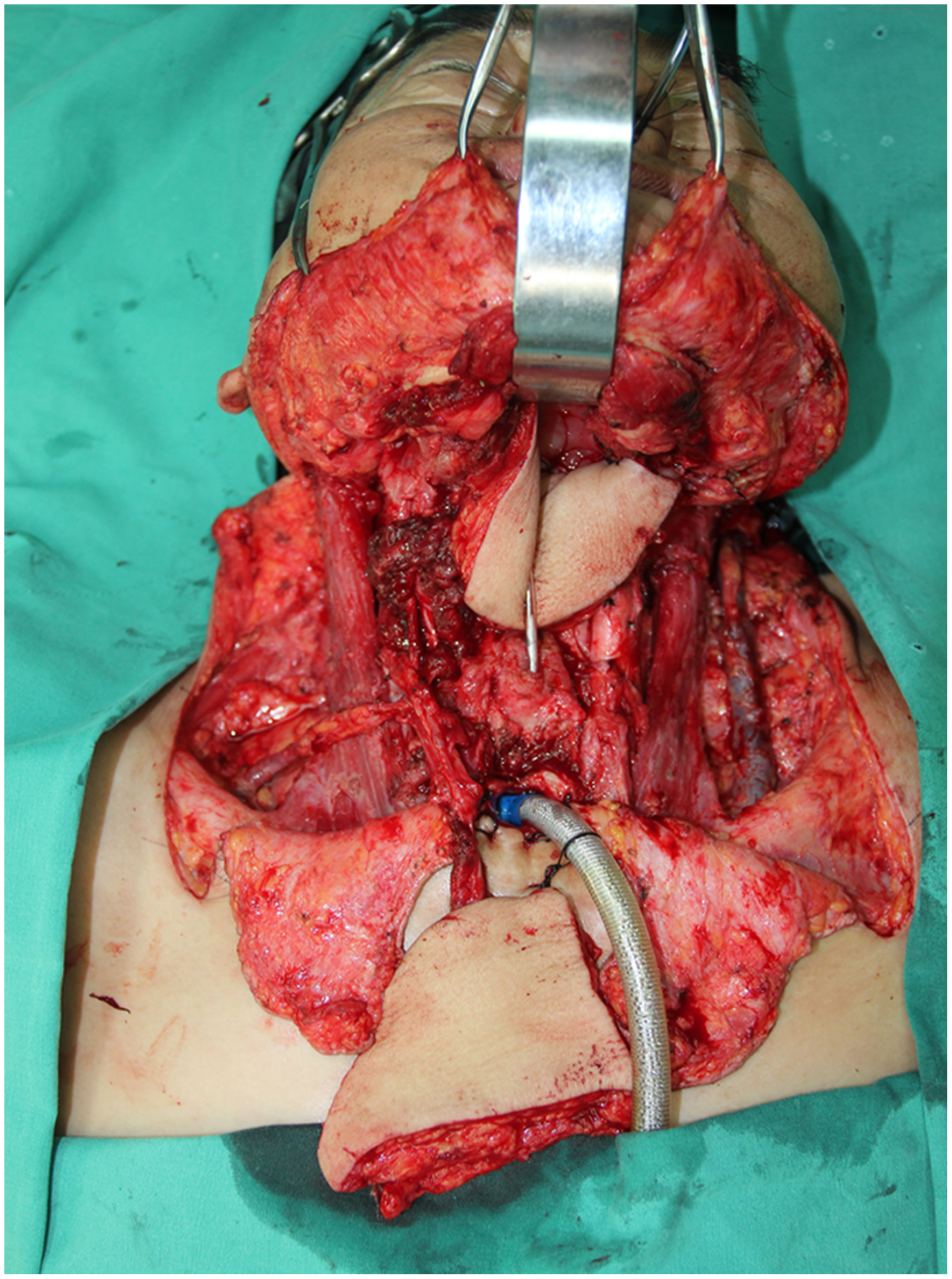
Intraoperative view of two separately skin paddles reconstructing neoesophagus and anterior neck skin respectively. The distal paddle with two separately perforators was tubularized by itself to form a neoesophagus.

**Figure 11:**
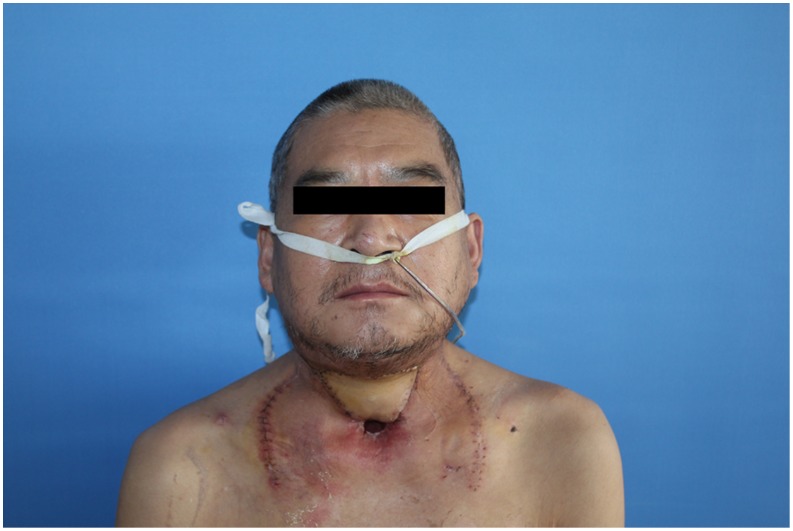
Appearance 14 days after operation with complete flap survival.

A 56-year-old male patient (case number 4 in Table 1) presented with a local recurrent hypopharyngeal cancer that invaded the esophagus and anterior neck skin (Fig. 7). A salvage resection involving the cancer, esophagus, anterior neck skin and bilateral neck dissection was performed. The resulting defects involved a part of circumferential esophagus and anterior neck skin (Fig. 8). A bipaddled ALT fasciocutaneous flap was harvested (Fig. 9). The distal paddle (7×12 cm^2^) was tubularized by itself to form a neoesophagus, while the proximal paddle (7×8 cm^2^) for the defect of anterior neck skin, which also could monitor the inner free flap (Fig. 10). The flap survived completely without any complications (Fig. 11). Patient had good functional outcome without fistula or stricture formation. However, this was the only one patient who complained of fatigue while ascending and descending stairs for 2 months after operation, and felt obvious recovery at 4 months after operation.

#### Case 3

**Figure 12:**
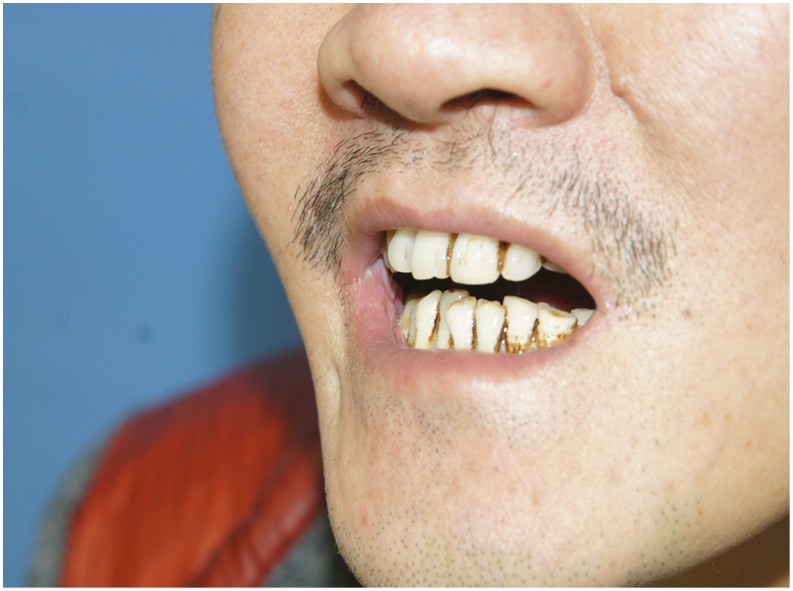
Preoperative view of trismus due to right buccal cancer and left OSF. The preoperative month opening was severely limited.

**Figure 13:**
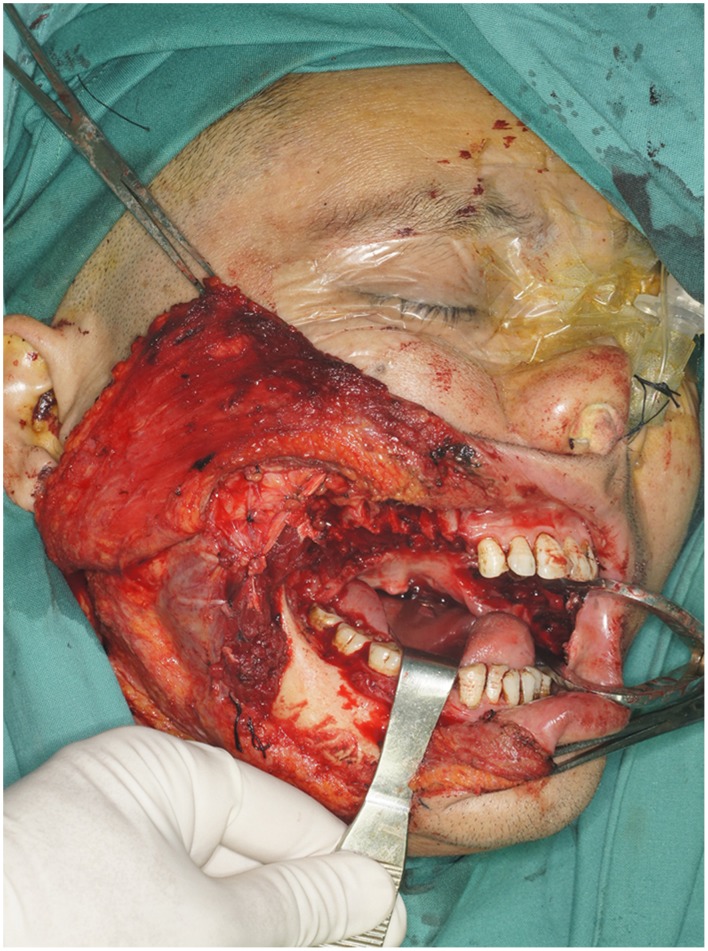
Intraoperative view of right full-thickness cheek defects and left buccal mucosa defect after the radical cancer surgery.

**Figure 14:**
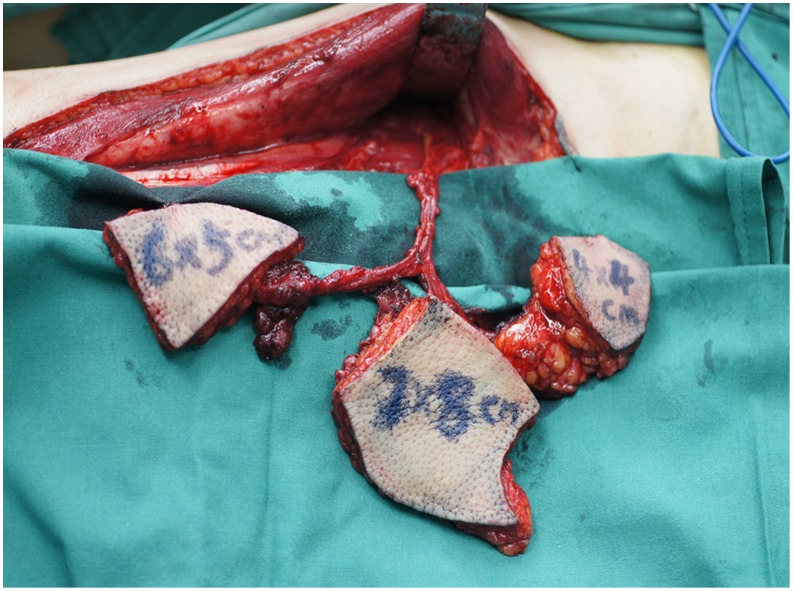
Intraoperative view after harvest of a tripaddled free ALT musculocutaneous flap.

**Figure 15:**
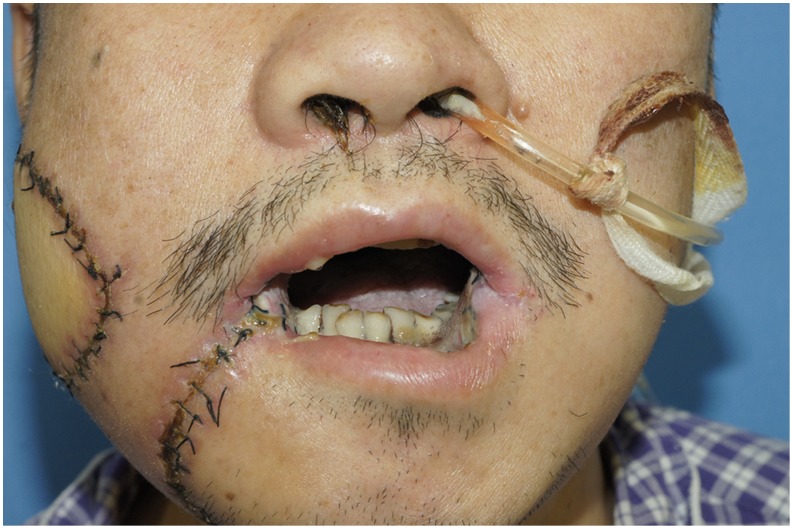
Early postoperative view of two proximal chimeric paddles restoring right full-thickness cheek defects and the distal paddle restoring left buccal defect. The postoperative month opening was obviously improved.

A 54-year-old male patient (case number 7 in Table 1) was presented with the right buccal tumor and progressive trismus of about 6-month duration which was caused by severe OSF (Fig. 12). The patient underwent a radical surgery for right buccal cancer, a transversal release of left OSF tissue, and right functional neck dissection. After ablation, the bilateral buccal defects were noted, including a left buccal mucosa defect (3×4 cm^2^), and a right through-and-through cheek defects which involved the intraoral defect (6×8 cm^2^) and the outer skin defect (4×6 cm^2^) (Fig. 13). A free ALT musculocutaneous flap with three independent skin paddles was harvested (Fig. 14). The distal paddle was used to resurface the left mucosa defect, and two remaining paddles were chimeric to reconstruct the right full-thickness cheek defects. The donor site was closed primarily. All skin paddles of the ALT flap were survival, and the postoperative month opening of the patient was improved obviously (Fig. 15).

## Discussion

There are several tissue organs with different locations and configuration in head and neck, and reconstruction of multiple and complex soft-tissue defects after radical surgery in this region demands a flap with three-dimensional requirement of both volume and surface. Two or more free flap transfers for complex defects in a single operation are frequently encountered, nevertheless there are several disadvantages of limited availability of recipient vessels, having to do two or more sets of microvascular anastomosis and a possible longer operating time.

Free ALT flap has become an important option for reconstruction of multiple anatomical locations in head and neck, which is often attributed to its multiple advantages: a long pedicle with good caliber, a large and pliable skin territory with the ability to design more than one skin paddle depending on the perforator anatomy, and the ability of two teams to work at the same time [10]. The most important advantage of ALT flap for multiple and complex defects is the fact that this flap can be harvested to include skin only or both skin and muscle, or as a chimeric flap with separately perfused skin paddles for multiple anatomical locations Longo et al [11]–[13] perfomed unipaddled or bipaddled free ALT perforator flaps for complex but adjacent defect locations in head and neck. However, there is few literature concerned on the reconstruction of more complex soft-tissue defects involving two or more nonadjacent anatomic sites. For such complex defects, ALT flap should be designed as multiple separately skin paddles to cover multiple defects in different anatomical regions simultaneously.

In our present series, all three types of complex soft-tissue defects are unsuitable to be reconstructed by an unipaddled free ALT flap with a single perforator, because there were at least two nonadjacent defect locations in one patient. Especially for type I patients presented with advanced buccal cancer and contralateral OSF, a radical ablation of buccal cancer was primary and imperative, however it was also necessary to release contralateral OSF buccal mucosa and correct the trismus [14]. OSF is a chronic oral mucosal disease primarily affecting oral cavity and always leads to the restriction of mouth opening, eventually impairing the ability to eat, speak and dental care [15]. For these OSF cases, a free multipaddled ALT flap should be designed to recover bilateral buccal defects, including a full-thickness cheek defects in one side and contralateral buccal defect. However, for cases with type III defect, a single free ALT flap with a single perforator might be a supplementary option for cases with no enough Intraoperative perforator by de-epithelializing the skin between the two paddles like the method introduced by Tan et al [16], Nevertheless, overmuch folding and looping of one flap can always increase the flap necrosis, while the design of individually perfused paddles with separately perforators can reduce the risk.

For the harvest of multipaddled free ALT flap, at least two separately and safe perforators based on descending branch of lateral circumflex femoral artery (dbLCFA) should be found intraoperatively on one thigh. However, one of principal uncertainties of free ALT flap is the variation of the perforator's anatomy and the occasional absence of satisfactory perforators. Kimata et al. [17] and Wei et al. [18] considered that 2 to 3 cutaneous perforators can be found to run through the medial edge of vastus lateralis. From our experience and the work of others, at least 1 suitable cutaneous perforator could be always found [19]. The first perforator with the largest diameter can be always found to exit within a 5-cm-diameter circle centered at the midpoint of the line between the anterior superior iliac crest and the superolateral border of the patella. Although 5–6% cases with absent cutaneous perforators were also described in some earlier studies, the small size of those presented perforators was considered as the likely reason for being overlooked [20], [21]. Although preoperative Doppler examination is commonly used to map out ALT cutaneous perforators, operator dependence and false positive are its main disadvantages. Shaw et al. [22] speculated that false positives represented either pure muscular perforators, or signals from the main descending branch because of their proximity to the tip of the probe when the intervening layer of fat is minimal. Chiu et al. considered that preoperative multi-detector computed tomographic angiography (CTA) could be performed to map perforator and evaluate the dominant vascularity in the suitable thigh before transferring chimeric ALT flaps [23]. However, it was hard for CTA to show the direct location of cutaneous perforators just like Doppler for surgeons. While in our enrolled cases, we can find at least 2 or 3 reliable cutaneous perforators intraoperatively on one thigh with the help of preoperative Doppler examination. So although there are many methods to locate the perforators, in our experience a handheld Doppler probe has adequate sensitivity for perforator location. Meanwhile, we agree with the free-style flap harvest concept, which means any cutaneous perforators located by a handheld Doppler probe can potentially be harvested by retrograde dissection as a free flap, regardless of regional anatomy. However, in our present study, for the only abandoned patient, who was an old female patient, it was a very rare case that we only found a single reliable cutaneous perforators on each thigh intraoperatively, although preoperative Doppler showed at least 2 perforators in one thigh The too small size of the presented perforators and moderate dystrophy of the case might be the main reasons. The more microsurgical experience and careful manipulation should be needed. However, for the rare patient in our present study, two unipaddled ALT flaps from both thighs had to be harvested to recover multiple defects in her head and neck.

In our present study, a small segment of vastus lateralis muscle around perforator was included in the majority of cases as additional volume to augment the dead space raised after radical cancer ablation and prevent tissue retraction of postoperative radiotherapy [19]. Meanwhile, we usually leave partial soft-tissue or a small deep fascial cuff around the perforator to prevent the vessel spasm, especially perforators and limit the risk of damage to the vessel. So the free ALT flaps in our cases were the musculocutaneous or fasciocutaneous flaps, not the perforator flap as classically described [24]. Free ALT musculocutaneous flap does not affect quadriceps muscular function, because only the vastus lateralis is dissected and still three bellies of the quadriceps can provide good functional synergy [25]. Meanwhile, careful preservation of the lateral cutaneous nerve and femoral motor nerve branches can decreases the risk of complications, such as lateral thigh paresthesia, musculoskeletal dysfunction, and compartment syndrome [26]. However, Anatomical variations include the nerve passing through the pedicle of the ALT flap, or passing between perforators, seen in 28% of a 36-human cadaveric thigh dissection study [27]. The close course of the vascular pedicle with the femoral motor nerve branch innervating the vastus lateralis could lead to nerve damage during flap elevation, resulting in knee extension weakness [28]. In our present study, only one patient (Case 2) complained of fatigue while ascending and descending stairs for 2 months after operation. This might be attributed to the intraoperative temporary damage of the femoral motor nerve branches in the vastus lateralis. Early physical therapy plays an important role in minimizing the weakness of the vastus lateralis [29]. However, in comparison with salvage reconstruction of the advanced cancer, this could be considered negligible.

Moreover, the selection of recipient vessel is also very important to avoid flap failure [30]. Although the vessels of the ALT flap match closely to the recipient vessels in head and neck, radical surgery of cancer in head and neck could usually cause the recipient vascular impairment or resection. There are two veins of different sizes accompanying the artery of the ALT flap, while many authors believed that only one accompanying vein anastomosis was enough for venous return [31]. Kimata et al [32] prefers to check the quality of venous back-flow and choose an appropriate vein for anastomosis after the anastomosis of the artery has been completed, because the flow strength of venous return sometimes differs between the two veins, unrelated to venous size. Rubino C [33] reported that flow rate measured postoperatively on flap arteries is significantly correlated with flap weight. In order to avoid congestion of the multiple ALT flap and postoperative complications, we usually chose a branch of internal jugular vein and the external jugular vein as the two recipient veins for anastomosis. It can secure the venous return of the flap and no flap congestion occurred in our series.

## Conclusion

In our experience, we believe that the multipaddled ALT chimeric flap is a reliable option for the reconstruction of complex soft-tissue defects with multiple different spatial orientations in head and neck, because it can provide several independent skin paddles for multiple separately defects simultaneously with minimal donor site morbidity.
